# Voriconazole-refractory fungal infection of phacoemulsification tunnel

**DOI:** 10.4103/0301-4738.67072

**Published:** 2010

**Authors:** Vikas Mittal, Ruchi Mittal, P C Sharma

**Affiliations:** Sanjivni Eye Care, Model Town, Ambala, Haryana, India; 1 Sharma Eye Hospital, Arya Chowk, Ambala, Haryana, India

**Keywords:** Fungus, microbial keratitis, patch graft, phacoemulsification, tunnel infection, voriconazole

## Abstract

A 44-year-old man presented 28 days after cataract surgery (phacoemulsification) in right eye with multiple pinpoint infiltrates in posterior stroma at cataract surgery wound site. Visual acuity was 20/60. Corneal scraping from the floor of the corneal tunnel revealed fungus which was later identified to be *Aspergillus flavus.* The patient was started on oral voriconazole 200 mg twice daily and topical voriconazole 1% every hour. Two intracameral injections of voriconazole (50 micrograms/ 0.1 ml) were given 72 h apart, five days after starting initial therapy. Infiltrates increased in size and density in spite of 20 days of voriconazole therapy. Full-thickness patch graft was done to arrest progressive necrosis. Four months after surgery, patient had 20/60 best-corrected visual acuity. There was no recurrence in one-year follow-up. Present case illustrates the therapeutic challenge in fungal tunnel infections and possibility of voriconazole-resistant *Aspergillus* species.

Self-sealing sutureless wounds are almost universal in modern cataract surgery. Published data on corneoscleral wound infections suggest both bacteria and fungus as etiological agents. Bacterial tunnel infections are reported to respond to medical therapy although surgical intervention may also be required.[1,2] Fungal infections of such incisions are especially difficult to treat because of poor corneal penetration of available antifungal agents and may have poor prognosis even after surgical intervention.[3] Voriconazole is a relatively new antifungal agent with good corneal penetration and shown to have promising results in ocular fungal infections.[4,6] Cases have been reported in which voriconazole has been successfully used in treatment of fungal keratitis including fungal tunnel infection.[6,7] We report a patient with a fungal infection of phacoemulsification corneal tunnel in a sutureless self-sealing wound that was refractory to topical and systemic voriconazole requiring patch graft as a therapeutic measure.

## Case Report

A 44-year-old gentleman was referred for irritation, watering and dimness of vision 28 days after cataract surgery (clear corneal phacoemulsification and intraocular lens implantation) in right eye. Records showed that he had uneventful cataract surgery with Best-Corrected Visual Acuity (BCVA) of 20/40 at first postoperative week. He was non-diabetic and there was no history of trauma after the surgery. Postoperatively, he was on topical prednisolone acetate 1% four times per day and topical ofloxacin 0.3% four times per day which he was continuing till his presentation to us.

**Figure 1:**
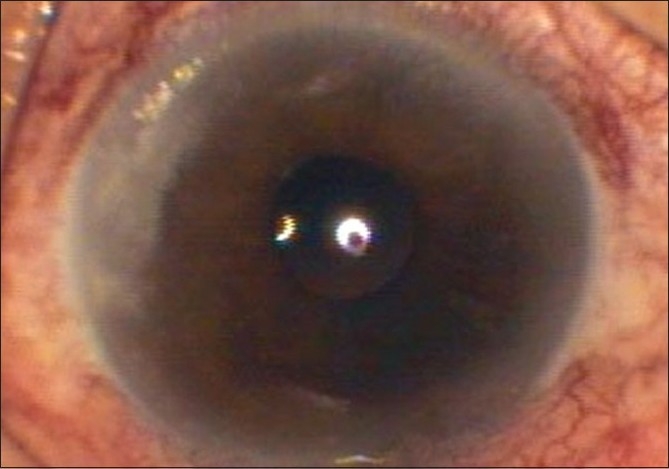
First presentation (28 days post phacoemulsification). Infiltrate: 2 mm × 3.4 mm; posterior stromal

He was seen in our clinic on the 28^th^ postoperative day (three days after the symptoms). BCVA was 20/60. Slit-lamp examination showed multiple pinpoint infiltrates in corneal tunnel area (2 mm × 3.4 mm) in posterior stroma [Fig. 1]. Siedel’s test was negative. Anterior chamber was quiet and fundus was within normal limits. Nasolacrimal duct was patent. Since infiltrates were in the posterior stroma only, corneal scrapings were taken from the floor of the tunnel and sent for Gram staining, 10% potassium hydroxide (KOH) mount and culture for fungus and bacteria. Gram stain and KOH mount didn’t show any organism. Since patient had developed these infiltrates 28 days after surgery, clinically it was decided to treat it like a fungal infection or slow-growing bacteria. Treatment given was topical voriconazole 1% one-hourly, moxifloxacin 5 mg/ml one-hourly and cycloplegics (1% atropine three times/day). A 1% solution of voriconazole was prepared by diluting 200 mg of the lyophilized intravenous preparation of voriconazole (Voritrop, INTAS Biopharmaceuticals) with 20 mL of normal saline. Once prepared, it was stored at 4 degrees and used for 48 h. Fungus (*Aspergillus flavus*) was grown on Sabouraud’s Dextrose Agar on the sixth day. Systemic voriconazole 200 mg twice daily was added to the treatment.

**Figure 2:**
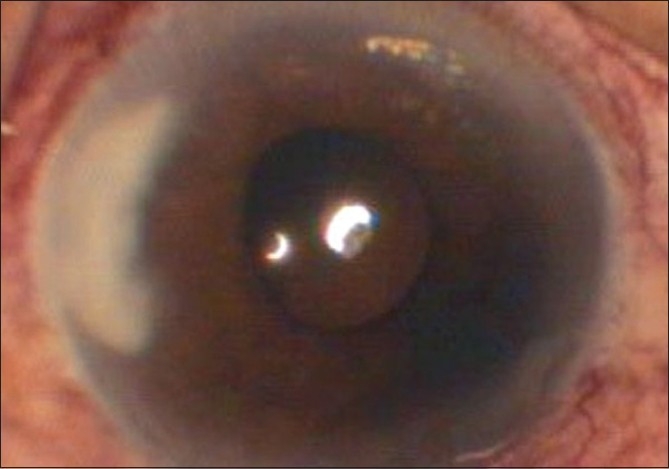
After five days of voriconazole therapy. Infiltrate: 2.6 mm ×4 mm; mid to posterior stromal

**Figure 3:**
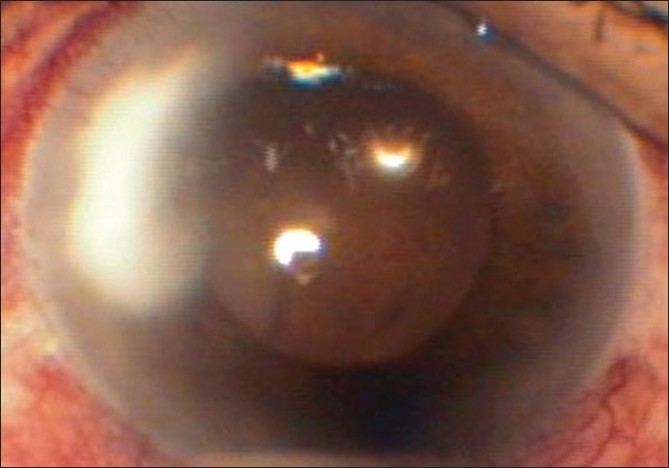
After 20 days of voriconazole therapy. Infiltrate: 3.1 mm X 4.5 mm; full stromal thickness

Infiltrates increased in density, size (2.6 mm × 4 mm) and depth (mid to posterior stroma) within five days of the above treatment [Fig. 2]. To ensure the full therapeutic dose at the site of inoculation of fungus (posterior stroma), two intracameral voriconazole (50 micrograms/ 0.1 ml) injections were given on Day 8 and Day 11 of presentation. Infiltrates increased to 2.8 mm X 4.1 mm and hypopyon appeared on Day 14. Topical natamycin 5% and amphotericin B 0.15% every one hour were added after epithelial debridement. Systemic voriconazole was continued for 20 days. Infiltrates kept on increasing with onset of tissue necrosis [Fig. 3].

Full-thickness therapeutic patch graft was done after 20 days of failed medical management. Infected and necrotic tissue was excised along with 2 mm surrounding clear cornea. Eleven mm trephine was used to make initial groove on the corneal side of the tissue to be excised which was cut by corneal scissors. Same diameter trephine was used to eccentrically punch the donor tissue. Limbal side of host and donor was hand-fashioned. Histopathology of excised tissue revealed fungal filaments in whole thickness of cornea. Half of the specimen that was sent for microbiological analysis revealed *Aspergillus flavus.*

**Figure 4:**
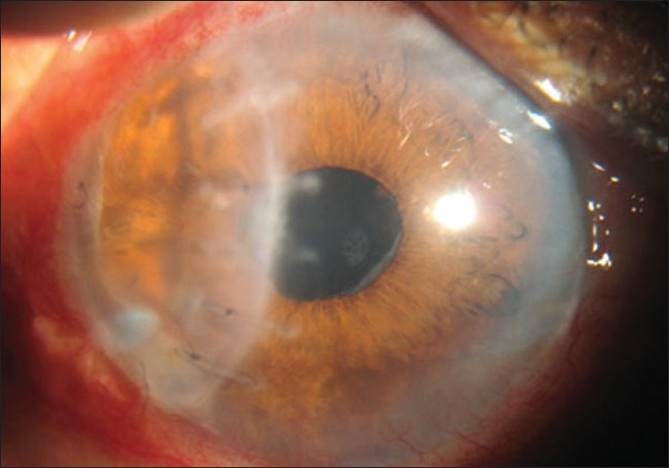
Four months after full-thickness patch graft

Postoperatively, patient was kept on topical natamycin 5%, atropine 1% and systemic ketoconazole 200 mg two times a day for three weeks after which topical steroids were started. Four months postoperatively patient had BCVA (-0.75/-2.0D@130degrees) of 20/60 [Fig. 4]. There was no recurrence of fungal infection in one-year follow-up period.

## Discussion

A review of ophthalmic literature in English language using PubMed revealed 11 cases of fungal wound infections after sutureless cataract surgery (manual small incision cataract surgery and phacoemulsification).[3,7–9] One out of two patients of Medicute *et al*. resolved on medical therapy (oral itraconazole 200 mg/day and topical 0.15% amphotericin B) while the other failed on the same treatment requiring lamellar keratectomy, scleral patch graft and limbal autograft.[8] The patient of Kehdi *et al*. did not respond to topical amphotericin B and systemic fluconazole and two perforating corneal grafts were required to save the eye.[9] Initial treatment started by Garg *et al*. included topical fluconazole 2%, topical amphotericin B 0.05% and systemic fluconazole for *Candida albicans* and topical natamycin 5% and systemic ketoconazole for *Aspergillus terreus.*[3] Only two cases responded to this treatment while the remaining five eyes progressed to phthisis bulbi. In 2007, Jhanji *et al*. reported a case of fungal tunnel infection (*Fusarium*) one month after clear corneal cataract surgery which resolved completely on topical and systemic voriconazole.[7]

Our case had fungal infection (*Aspergillus flavus*) of the main port which presented 28 days after clear corneal phacoemulsification and started with corneal infiltrates mainly in the posterior cornea. Out of all available antifungal agents, voriconazole has the broadest spectrum of antifungal activity and best corneal penetration which was required in our case considering the deep-seated infiltrates.[4,5,10,11] Further, apart from the case of Jhanji *et al,* there is enough ophthalmic literature which suggests that voriconazole is a powerful tool in the management of fungal keratitis and endophthalmitis.[12] Hence, it was decided to treat our case medically first with topical and systemic voriconazole.

Because the infiltrate was deep and not amenable to routine corneal scrapings, scrapings from the inner lip of the tunnel were taken under the microscope. No organism was found on potassium hydroxide 10% wet mount and gram stain. In view of late onset of infection, fungus and slow-growing bacteria were suspected and hence moxifloxacin was added to voriconazole. After having the culture report of *Aspergillus* (sixth day), antibiotic could have been stopped.

In tunnel infections, organism is inoculated in the potential space between floor and roof of tunnel and may gain early access to the anterior chamber and vitreous cavity, giving rise to endophthalmitis.[3] To ensure the best prophylaxis against the same and to ensure maximum therapeutic concentration at site of organism inoculation (deep stroma), intracameral voriconazole was given in a dose described for intravitreal route (50 micrograms/0.1 ml). No adverse effects in the form of either increased anterior chamber reaction or corneal edema were noted.

Even after giving voriconazole from all possible routes, infiltrates in our case kept on increasing and required surgical intervention to save the globe. Failure of voriconazole therapy for fungal keratitis has been reported in infections with *Fusarium* and *Colletotrichum* species.[11,13] Present case was post cataract surgery fungal keratitis caused by *Aspergillus* species which failed to respond to voriconazole.

One of the reasons of poor prognosis of fungal tunnel infections is use of corticosteroids before the onset of clinical signs of infection which may cause the infecting organisms to spread diffusely. Our case was also on topical steroids before onset of infection.

Surgical keratectomy debulks the infected tissue, contributing to the rapid resolution of the infection. One major concern after keratectomy or patch graft in terms of wound infection may be the possibility of alterations of the corneal topography. However, this may not be clinically evident, if surgical intervention is done early while infiltrates are still localized to the periphery. Our case was managed by patch graft and the final outcome was satisfactory.

Sterilizing technique of the cataract surgeon was looked into to have information on possible source of infection. There was reuse of metallic blades after overnight exposure in a formalin chamber. And, there were no associated cluster cases. Contaminated surgical instruments have been suggested to be the source of infection in such cases and our case strengthens this suggestion.[14]

The present case highlights the role of early surgical intervention in fungal tunnel infections and the possibility of voriconazole-resistant *Aspergillus* species.
